# Family Physicians’ Use of, Barriers to, and Attitudes Toward Remote Diagnosis and Treatment in China: A National Web-Based Survey

**DOI:** 10.3390/healthcare13050481

**Published:** 2025-02-23

**Authors:** Yao Tang, Lianjun Li, Wanyue Dong, Hong Xie, Yiwei Qiu, Ruhai Bai

**Affiliations:** 1 School of Marxism, Xi’an Jiaotong University, Xi’an 710049, China; tangyao@stu.xjtu.edu.cn; 2 School of Public Affairs, University of Science and Technology of China, Hefei 230026, China; lianjunli@ustc.edu.cn; 3 School of Elderly Care Services and Management, Nanjing University of Chinese Medicine, Nanjing 210023, China; wanyuedong@njucm.edu.cn; 4 The Digital Medicine Society of the Shanghai Medical Association, Shanghai 200040, China; xiehong1969@163.com; 5 School of Public Affairs, Nanjing University of Science and Technology, Nanjing 210094, China; yiweiqiu@njust.edu.cn

**Keywords:** family physician, telehealth, digital health, remote diagnosis and treatment, usage, barriers, attitudes, primary health care, China

## Abstract

**Background/Objectives:** China has introduced a series of policies to encourage family physicians (FPs) to provide remote diagnosis and treatment (RDT) services, with the ultimate goal of providing more continuous, convenient, and efficient medical services for all residents, especially elderly patients and patients with chronic diseases. However, few studies have focused on this important issue. The aim of this study was to provide a comprehensive description of FPs’ use of, barriers to, and attitudes toward RDT in China. **Methods:** A cross-sectional survey was implemented for this study. The data were analyzed via basic descriptive statistics and are expressed as percentages. Additionally, a single-factor logistic regression was used to compare groups in terms of outcome measures. **Results:** Among the 682 respondents, 63.8% had participated in RDT, and 36.2% had never participated in RDT. Among the 435 respondents who participated in RDT, 19.1% were high-frequency users, and 80.9% were low-frequency users. The results of the single-factor logistic regression revealed that there were significant differences in the use of RDT among FPs in terms of age (*p* = 0.034), educational background (*p* < 0.001), hospital type (*p* = 0.008), income as a result of their work as an FP (*p* = 0.002), form of employment (*p* = 0.001), and general practitioner status (*p* < 0.001). Moreover, there were significant differences in the use frequency of RDT among FPs in terms of age (*p* = 0.009), years of practice as a health service provider (*p* = 0.001), years of practice as an FP (*p* = 0.003), educational background (*p* = 0.048), and working hours as an FP (*p* = 0.014). However, a lack of policy support (58.5%), technology support (55.3%), and information support (52.5%) were the top three factors hindering FPs from participating in RDT. Overall, FPs had positive attitudes toward RDT services, with more than half of the respondents expressing that they could benefit from such services and showing increasing interest in using them. **Conclusions:** The findings of this research can improve policymakers’ understanding of FPs’ use of, barriers to, and attitudes toward RDT. Our findings also provide suggestions, such as those for improving the promotion of RDT, optimizing relevant laws, and providing technical support for FPs to use RDT, which may help optimize related policies. There are also some limitations in our study; for example, the sample in this study included provincial administrative regions across China, but not all provinces were covered. In the future, research covering all provinces of the country could be carried out to make the research more nationally representative.

## 1. Introduction

Family physician services (FPSs) refer to physicians or general practitioners (GPs) trained to provide primary and continuing care, such as health checkups, consultations, and chronic disease management, for all inhabitants and to arrange for other health specialists to provide related healthcare services if needed [1]. In the 1960s, FPSs began in Western countries [2,3]. Later, as an important policy tool to achieve the ambitious goal of “primary health care for all” proposed by the Alma-Ata Declaration [4], FPSs constituted a crucial component of primary healthcare (PHC) [1,5] and were strongly promoted worldwide. To date, FPSs have been introduced in more than 50 countries [6], such as the United States [7], France [8], the Netherlands [9], and Indonesia [10]. In recent years, especially after the outbreak of COVID-19, the use of innovative internet medical systems, including a range of online medical services, such as medical advice, consultations, and prescriptions, has accelerated greatly in PHC worldwide [11]; for example, countries such as the United States [12], the UK [13], Canada [14], the Netherlands [9], and Singapore [15] actively introduced remote healthcare services as PHC services. China is no exception to this trend [16].

China has made remarkable achievements in strengthening its FPS system in recent years [17]. However, the system faces challenges in providing health care services for China’s population, which represents one-fifth of the world’s population, is aging, and has a growing prevalence of chronic noncommunicable diseases [17]. However, remote diagnosis and treatment (RDT) services can overcome the limitations of geographical boundaries, providing accessible health services [18]. In this context, the National Health Commission (NHC) has called for family physicians (FPs) to provide RDT services with the ultimate goal of providing more continuous, convenient, and efficient medical services for all residents, especially elderly individuals, patients with chronic diseases, and residents in remote areas [19,20,21]. For example, in April 2019, the NHC issued the National Standards and Specifications for the Informatization Construction of Grassroots Medical and Health Institutions, emphasizing the need to vigorously promote “FP + RDT services” [19]. In March 2020, the NHC issued the Notice on the Classified and Accurate Work of Grassroots Medical and Health Institutions in the Prevention and Control of COVID-19, indicating that FPs should be encouraged to innovate service models to optimize service processes and improve service efficiency [20]. Overall, in recent years, China has introduced new policies to promote FPs’ use of RDT, indicating the importance the Chinese government attaches to this issue.

In general, the majority of studies related to FPSs have been conducted around traditional FPSs, focusing on coordination mechanisms with health insurance [22], the development and current situation of FPSs [23,24], factors influencing users’ attitudes toward FPSs [1,6,25,26], and the impacts of FPSs [27]. However, the important issue of FPs’ use of RDT has not received sufficient attention, and the majority of the limited related studies have been conducted in developed countries, mainly North America and Europe [11]. Concerning FPs’ use of RDT in developing countries, the experience of China, as the largest developing country in the world, would provide more useful and practical evidence for countries with similar contexts than studies based on developed countries. Therefore, it is meaningful to disseminate such information from China internationally.

The aim of this nationwide study was to answer the following questions: (1) the overall situation of FPs’ use of RDT, including whether they have ever used RDT and the frequency with which they have provided RDT services; (2) demographic differences in FPs’ provision of RDT services; (3) factors hindering them from providing RDT services; and (4) attitudes toward RDT services.

## 2. Materials and Methods

### 2.1. Research Design and Measurement Instrument

**Figure 1:**
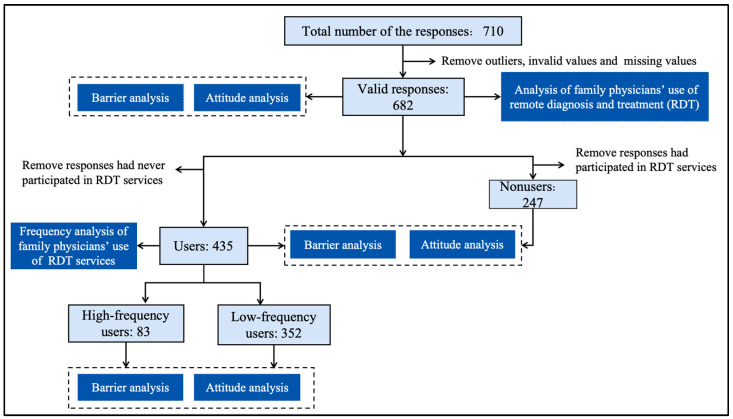
Research process.

Figure 1 shows the details of the study design and research process for this study: (1) the overall situation of FPs’ use of (including users, nonusers, high-frequency users, and low-frequency users), barriers to, and attitudes toward RDT were analyzed; (2) FPs’ barriers to and attitudes toward RDT were analyzed in terms of whether they had ever used RDT services; (3) for the FPs who have used RDT services, FPs’ use of, barriers to, and attitudes toward RDT were analyzed in terms of high-frequency users and low-frequency providers of RDT services.

The measurement instrument was designed as a four-part questionnaire that included 36 questions. The first part was used to collect the respondents’ basic demographic information and included 14 questions. The second part addressed the current situation of the FPs’ participation in RDT services and included 2 questions: (1) whether the respondents had ever used RDT; and (2) the frequency with which they provided RDT services for their patients per week. The third part examined the barriers hindering them from providing RDT services and included 13 questions. The fourth part assessed the respondents’ attitudes toward RDT and included 3 questions.

This study was reviewed and approved by the Medical Ethics Committee of Chinese PLA General Hospital with the approval numbers S2024-169-01 and 2024-1-4. In addition, before the participants participated in this questionnaire survey, we explained the aims of our research and declared that the survey data would be used only for the current study and that personally identifiable information would be collected. All participants gave their informed consent for inclusion.

### 2.2. Data Collection

FPs from the Chinese Medical Doctor Association (CMDA), a national-level social organization, were selected as the research subjects. In total, 710 respondents from 21 provincial administrative regions and 59 cities across China participated in this questionnaire survey. We sent the questionnaire to the target participants through the web-based survey tool Wenjuanxing, which is the largest questionnaire survey website in China. From March 29th to April 10th, 2024, we sent the Wenjuanxing survey link to WeChat groups. Mobile internet protocol addresses were used to ensure that individuals completed the questionnaire only once.

Data analysis was conducted using IBM SPSS Statistics 27 (IBM Corp., Armonk, NY, USA). *p* values < 0.05 were considered statistically significant. First, the data were analyzed via basic descriptive statistics and are expressed as percentages. Single-factor logistic regression was used to compare groups in terms of outcome measures.

## 3. Results

### 3.1. Characteristics of the Respondents

As of the end of 2021, China had approximately 1.435 million FPs, forming 431,000 teams to provide contracted services to residents [28]. A total of 710 responses were received from Wenjuanxing. Among the 710 responses, 28 were eliminated for the following reasons: (1) the respondent was not an FP; (2) the responses contained exactly the same answers to all the questions; (3) an obvious logical contradiction was found in the responses; (4) all the questions were answered in only a few seconds; or (5) the responses had missing values. Thus, a total of 682 responses were included.

**Figure 2:**
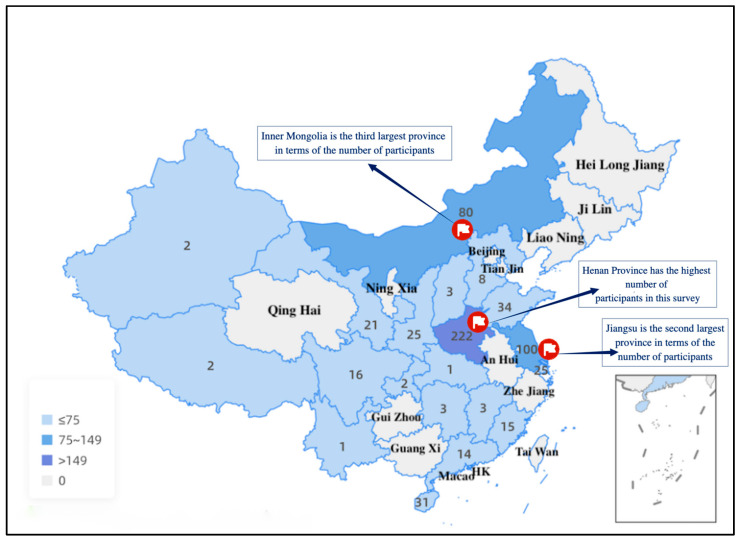
Distribution of the family physician sample in China by province. The numbers represent how many questionnaires were collected in each corresponding province.

As shown in Figure 2, a total of 682 FPs from 21 provinces in China participated in this survey. The characteristics of the participants are shown in Table 1. Among the 682 participants, 43.0% were male, and the participants had a median age of 37 years old. The median number of years of practice the respondents had worked as health service providers was 12, and the median number of years of practice the respondents had worked as FPs was 4, respectively. Overall, 49.6% of the participants had a Bachelor’s degree. Among them, 46.0% were from rural areas (township hospitals), and 32.4% were from first-tier cities. With respect to employment status, 45.7% of the respondents held positions within establishments. Among the 682 respondents, 91.5% were part of an FP team. The median number of total working hours on weekdays was 8, and the median number of working hours related to FP work was 4. With respect to income, 39.4% of the respondents earned between 280.8 and 421.1 USD after tax per month, and 43.4% of the respondents indicated that their income related to working as an FP was less than 140.4 USD per month. Table 1 also shows the overall situation of the FPs’ use of RDT. Among the 682 participating FPs, 63.8% had provided RDT services. Among the 435 FPs who had provided RDT services, 80.9% used RDT fewer than seven times a week. 

### 3.2. Use of RDT in Terms of FP Demographic Characteristics

**Figure 3:**
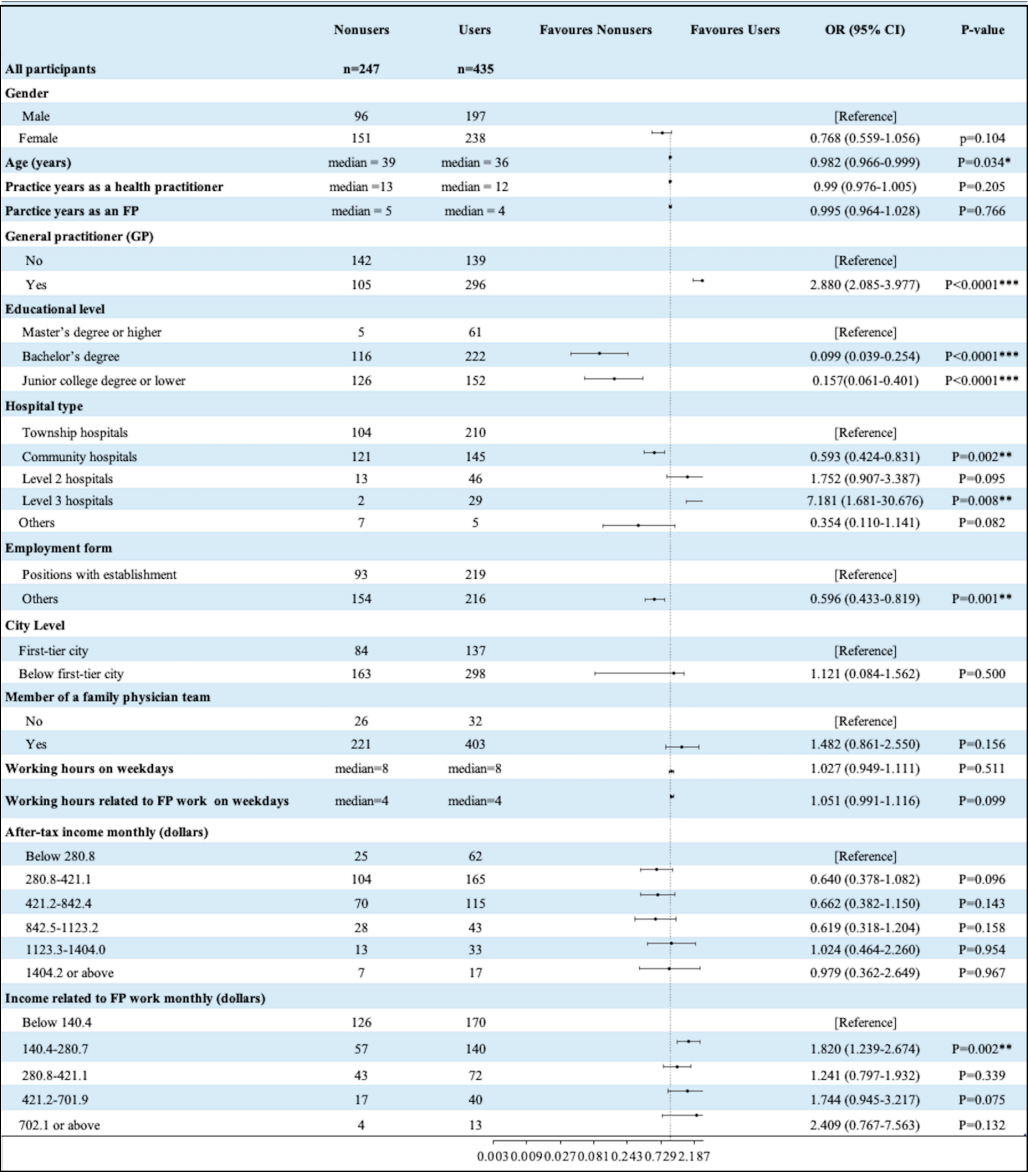
Results of single-factor logistic regression in terms of the use of RDT. Notes: * (*p* < 0.05), ** (*p* < 0.01), *** (*p* < 0.001).

In this section, we explore the demographic differences in FPs’ provision of RDT services from the perspective of whether they provide RDT services and the frequency with which RDT services are provided. As shown in Figure 3, a single-factor logistic regression was carried out among the 682 practitioners, with RDT as the dependent variable and their demographic characteristics as the independent variable. According to the research results, compared with older FPs, younger FPs were more likely to provide RDT services (OR = 0.982, *p* = 0.034). Compared with non-GPs, GPs were more likely to provide RDT services (OR = 2.880, *p* < 0.0001). With respect to educational background, compared with respondents with a Bachelor’s degree, those with a college degree or below were less likely to provide RDT services, and those with a graduate degree or PhD were more likely to do so (OR = 0.099, *p* < 0.0001; OR = 0.157, *p* < 0.0001). In terms of medical institutions, compared with respondents from township hospitals, respondents affiliated with Level 3 hospitals were more likely to provide RDT services (OR = 7.181, *p* = 0.008). With respect to employment status, compared with respondents with an official position, respondents with no official position were less likely to provide RDT services (OR = 0.596, *p* = 0.001). Additionally, those with incomes as a result of their work as an FP ranging from 137.0 to 273.8 USD were more likely to provide RDT services than respondents with a monthly income of less than 137.0 USD (OR = 1.820, *p* = 0.002). However, whether FPs used RDT did not differ significantly in terms of sex, years of practice (including years of practice as a health care provider and years of practice as an FP), geographical area (urban area or rural area), city level, membership on an FP team, working hours, or overall after-tax income (see Figure 3).

**Figure 4:**
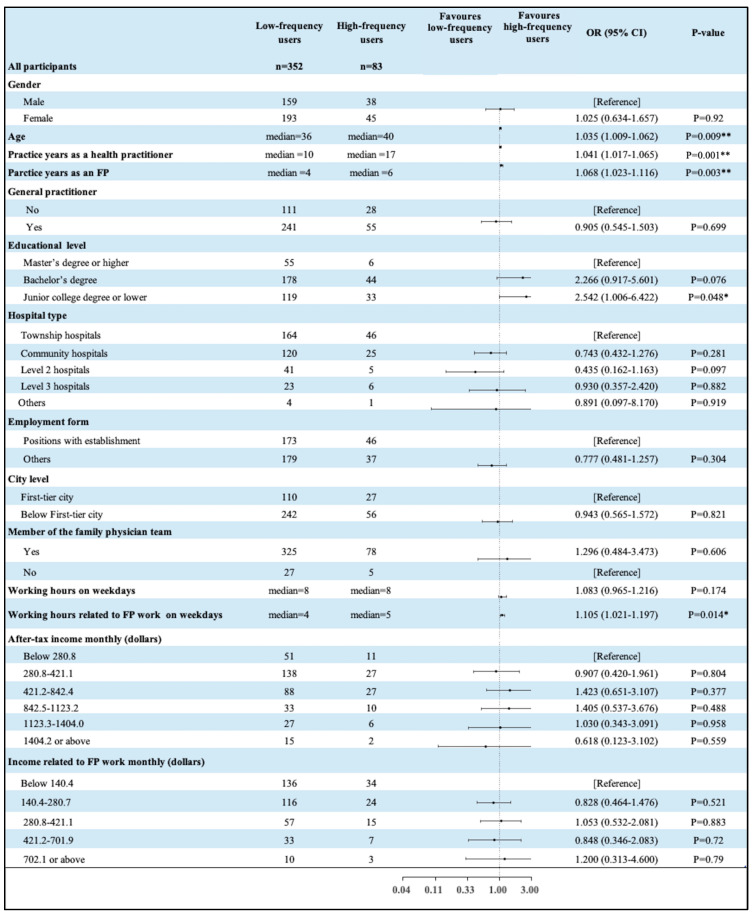
Results of single-factor logistic regression in terms of the frequency of RDT use. Notes: * (*p* < 0.05), ** (*p* < 0.01).

A single-factor logistic regression was subsequently carried out among the 435 practitioners, with the frequency of their use of RDT as the dependent variable and their demographic characteristics as the independent variable. As shown in Figure 4, among the 435 FPs who had used RDT, compared with younger FPs, older FPs were more likely to provide RDT services frequently (OR = 1.035, *p* = 0.009), and practitioners with more years of practice, including practice as a health care provider and practice as an FP, were more likely to provide RDT services frequently (OR = 1.041, *p* = 0.001; OR = 1.068, *p* = 0.003, respectively). In terms of educational background, compared with respondents with a graduate degree or PhD, respondents with a college degree or below were more likely to provide RDT services frequently (OR = 2.542, *p* = 0.048). For working hours related to RDT, respondents who spent more time in FPSs were more likely to provide RDT services frequently (OR = 1.041, *p* = 0.001). However, there was no significant difference in the frequency of FPs’ provision of RDT services in terms of sex, GP status, hospital type, employment status, city level, FP team membership, working hours on working days, or income (see Figure 4).

### 3.3. Factors Hindering FPs from Providing RDT Services

In this section, we analyze the factors hindering FPs from providing RDT services from the perspective of all responders, RDT users, RDT nonusers, high-frequency RDT users, and low-frequency RDT users. As Table 2 shows, the top three problems related to the use of RDT were as follows: traditional, established habits regarding medical treatment are difficult to change (71.1%), the publicity and promotion of RDTS are insufficient (63.7%), and the facilities of medical institutions are insufficient (61.7%). In addition, the top three factors that hinder FPs from providing RDT services are as follows: the lack of policy support (58.5%), the technology applied in RDT is not mature enough (55.3%), and residents do not understand RDT (52.5%).

**Figure 5:**
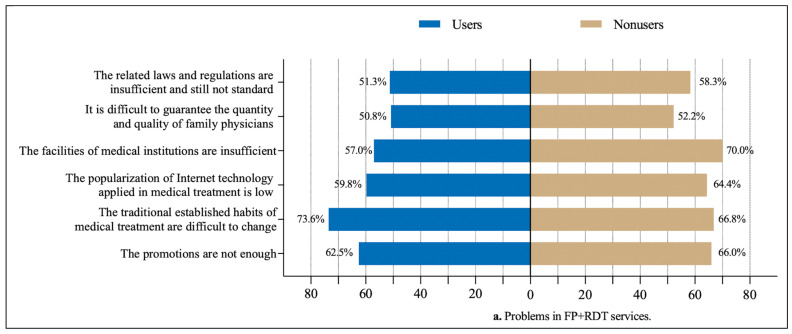

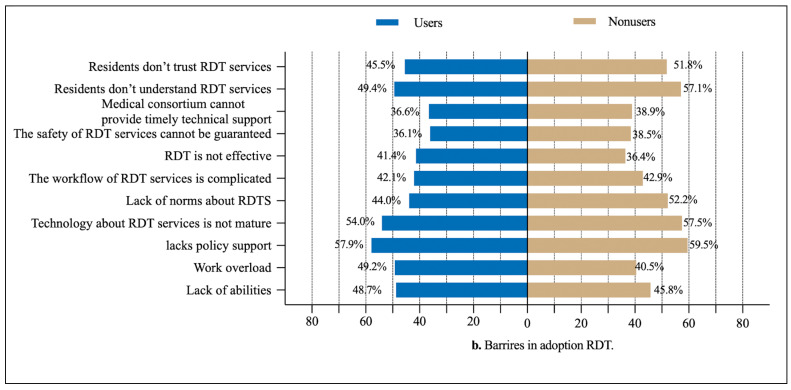
Factors hindering FPs from participating in RDT in terms of users and nonusers (n = 682).

Furthermore, as shown in Figure 5, for the FPs who had used RDT, they indicated that traditional, established habits regarding medical treatment are difficult to change (73.6%), which was the greatest problem in using RDT, whereas for the FPs who had never applied internet technologies in FPSs, they indicated that the facilities of medical institutions are insufficient (70.0%), which was the greatest problem in using RDT (see Figure 5a). The lack of policy support was the most significant factor hindering both users and nonusers from providing RDT services (57.9% and 59.5%, respectively) (see Figure 5b).

**Figure 6:**
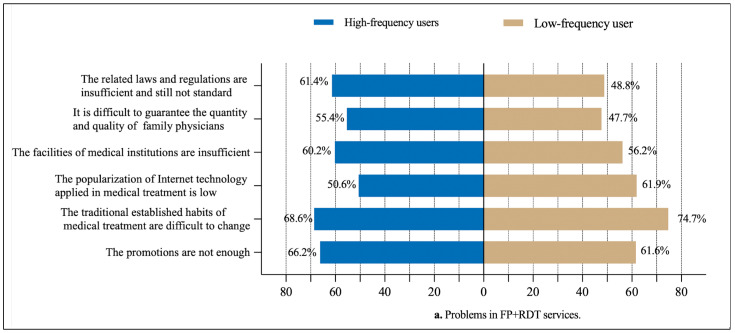

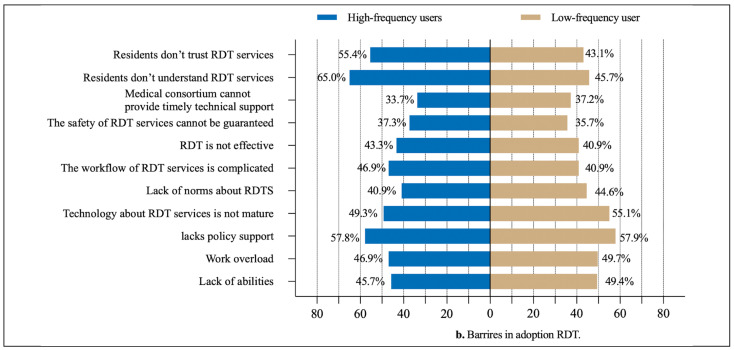
Factors hindering FPs from participating in RDT in terms of high-frequency users and low-frequency users (n = 435).

Furthermore, as shown in Table 1, among the 435 (63.8%) FPs who had provided RDT services, 352 (80.9%) were low-frequency users, and 83 (19.1%) were high-frequency users. For both groups, they indicated that the greatest current problem regarding the use of RDT in China was that traditional established habits regarding medical treatment are difficult to change (68.6% and 74.7%, respectively) (see Figure 6a). With respect to the barriers that prevented them from using RDT, for low-frequency users, the lack of policy support (57.9%) was the greatest barrier; however, for high-frequency users, the greatest barrier was that residents do not understand RDT (65.0%) (see Figure 6b).

### 3.4. FPs’ Attitudes Toward RDT

**Figure 7:**
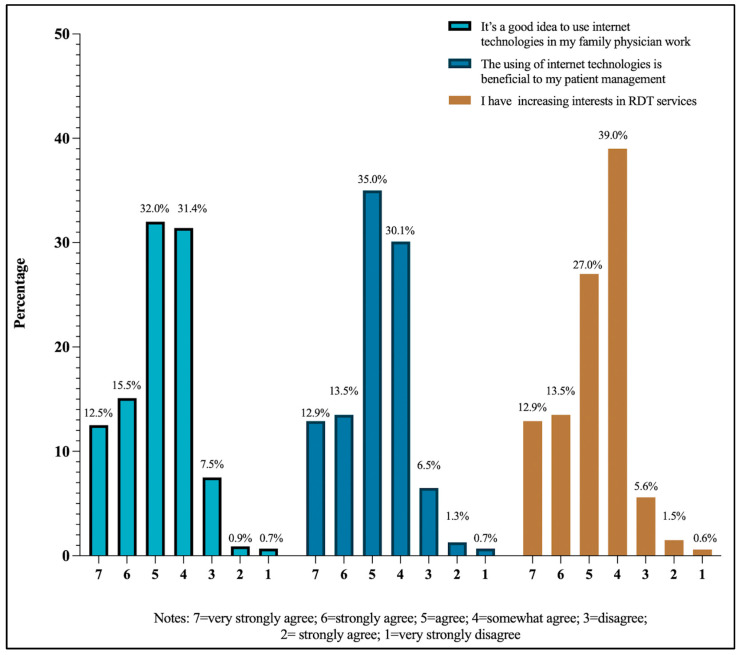
FPs’ attitudes toward RDT (n = 682).

In this section, we analyze FPs’ attitudes toward RDT services from the perspective of all responders, RDT users, RDT nonusers, high-frequency RDT users, and low-RDT users. As shown in Figure 7, overall, FPs had positive attitudes toward RDT. In response to the statement that it is a good idea to use internet technologies in my FP work, 32.0% of the participants agreed, 15.1% of the respondents strongly agreed, and 12.5% of the respondents very strongly agreed. Regarding the statement that the use of internet technologies is beneficial to their patient management, 35.0% of the participants agreed, 13.5% of the respondents strongly agreed, and 12.9% of the respondents very strongly agreed. Moreover, more than half of the respondents expressed increasing interest in using RDT in their future work.

**Figure 8:**
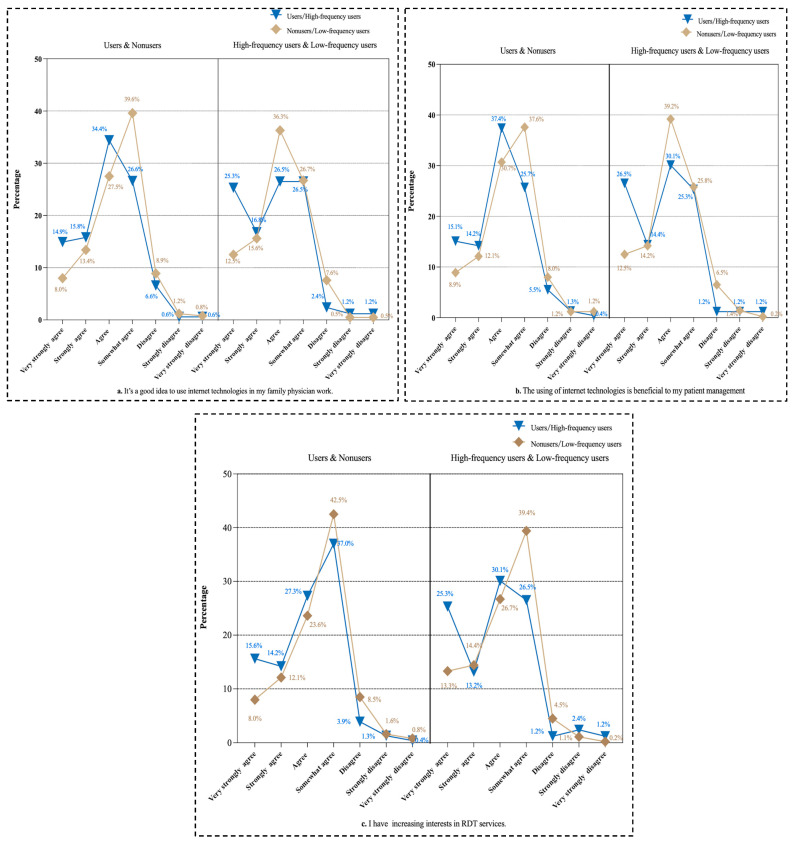
FPs’ attitudes toward RDT in terms of users and nonusers (n = 682) and high-frequency users and low-frequency users (n = 435).

As shown in Figure 8, respondents who had used RDT and high-frequency users had more positive attitudes toward RDT than the others did. A total of 14.9% of the users and 25.3% of the high-frequency users very strongly agreed that it is a good idea to use internet technologies in their work related to FPS; however, only 8.0% of the nonusers and 12.5% of the low-frequency users very strongly agreed. In response to the statement that using internet technologies is beneficial to their patient management, 15.1% of the users and 26.5% of the high-frequency users strongly agreed, whereas only 8.9% of the nonusers and 12.5% of the low-frequency users strongly agreed. In addition, 15.6% of the users and 25.3% of the high-frequency users showed very strong interest in using RDT in their future work, but only 8.0% of the nonusers and 13.3% of the low-frequency users showed very strong interest in the use of RDT (see Figure 8).

## 4. Discussion

Unlike previous studies focused on the use of RDT in developed countries, to our knowledge, our research is the first to analyze the RDT service provision of FPs from 59 major cities in China, and our findings largely reflect the current situation of FPs’ participation in RDT in China. In addition, we analyze FPs’ attitudes toward and barriers to using RDT from the perspective of users, nonusers, high-frequency users, and low-frequency users and offer specific suggestions on the basis of our findings.

### 4.1. Principal Findings

#### 4.1.1. The Use of RDT Among FPs

By May 2023, more than 420,000 FP teams had been established nationwide, offering healthcare services to contracted residents, particularly key groups such as patients with chronic diseases and elderly individuals [29]. In total, 682 of them participated in this research. Among the 682 participants, 435 (63.8%) had used RDT. Compared with research conducted in developed countries, such as the United States [30] and the Netherlands [9], the rate of RDT utilization among FPs in China was relatively low. We believe that the possible reasons for this phenomenon may be as follows: first, although the government promotes telemedicine, China’s FPSs started relatively late, and the policies and standardization systems in China are not well developed compared with those of developed countries, with a lack of clear operational guidelines and regulatory mechanisms; second, promotional efforts have been insufficient, and the level of awareness of internet-based medical consultations among residents and FPs is relatively low; and third, FPs in China usually face a heavy workload, leaving them with limited time and energy to also engage in telemedicine. However, more comprehensive explanations for this phenomenon still need to be further studied.

In addition, we also found that the use of RDT among FPs from Level 3 hospitals was greater than that among FPs from township hospitals. One of the possible reasons may be the uneven distribution of medical resources in China. Level 3 hospitals refer to large hospitals that provide high-level medical services for multiple regions and are usually equipped with advanced medical facilities. However, township hospitals are an essential part of the rural healthcare system, are located in rural areas, and primarily serve rural residents. Compared with township hospitals, Level 3 hospitals are equipped with advanced medical equipment and well-educated medical staff, which are crucial for determining doctors’ use of online services [12,31,32].

One of the most interesting findings in our research is that, compared with older FPs, younger FPs were more likely to provide RDT services. However, with respect to the frequency of RDT utilization, older FPs presented a higher use frequency than younger FPs. We argue that the possible reasons may be as follows: on the one hand, in general, younger doctors are more familiar with internet technology and have fewer technical barriers to using RDT than older doctors; thus, their utilization rate is usually higher. Older doctors are at high risk of facing technical barriers; therefore, their utilization rate is lower. On the other hand, older FPs usually face greater financial burdens, such as raising children and taking care of their parents. Thus, considering the income brought by the provision of RDT services, older FPs are more likely to frequently use RDT. The same reasoning can explain why doctors with more years of practice were more likely to frequently use RDT.

Another interesting finding in our research is that FPs with higher education and degrees were more likely to provide online services; however, FPs with lower education and degrees were more likely to be high-frequency users. We believe that the possible reasons for this phenomenon may be as follows: first, generally, highly educated FPs have better knowledge of policies related to family physicians, and they can make use of these policies and build a better career path by participating in FP policies [25]. Second, highly educated doctors are likely to work in high-level hospitals that are equipped with advanced medical equipment, which enables them to provide RDT services for patients. However, working in high-level hospitals entails a high amount of pressure on FPs’ daily work, which could reduce the time they spend providing FPSs. Finally, highly educated doctors usually have higher salaries than less educated doctors; thus, highly educated doctors may not be as attracted to the income brought by providing RDT services and are less likely to use RDT with a high frequency. In addition, according to our research, GPs provided RDT services at a higher frequency than other doctors did. Doctors with established positions and higher incomes as a result of their work as an FP were more likely to use RDT. These findings are in line with those of most previous studies [6,25,26,33].

#### 4.1.2. FPs Participating in RDT Services Have Great Development Potential in China

The survey results show that there are still many obstacles that hinder FPs from providing RDT services, such as the following: traditional, established habits regarding medical treatment are difficult to change, the publicity and promotion of RDTS are insufficient, and the facilities of medical institutions are insufficient. However, overall, FPs in China are shifting towards a positive attitude toward RDT. The vast majority of the FPs who participated in the survey agreed that the use of internet technologies is beneficial to patient management and expressed increasing interest in using RDT in their future work. Moreover, we also found that FPs have had good experiences participating in RDT services because, compared with nonusers and low-frequency users, FPs who have participated in RDT services, especially high-frequency users, have a more positive attitude toward RDT. The theory of planned behavior (TPB) has fully demonstrated a correlation between attitudes and behavioral intentions. In the field of remote healthcare research, many studies have shown that users’ positive attitudes are significantly positively correlated with their participation in remote diagnosis and treatment and that the more positive their attitudes are, the greater the behavioral intentions of those participating in remote diagnosis and treatment [34,35]. Overall, FPs’ positive attitudes toward remote diagnosis and treatment have laid a foundation for the further promotion and adoption of remote diagnosis and treatment in China.

#### 4.1.3. Suggestions to Promote the Use of RDT Among FPs

(1) Improving the promotion of RDT

According to our research, the insufficient promotion of RDT (63.7%) was one of the greatest problems regarding FPs’ use of China’s RDT services system. Moreover, approximately 52.5% of the FPs reported that residents’ lack of understanding regarding RDT was a barrier preventing them from using RDT. As dozens of previous studies have suggested, the lack of information regarding aspects such as related policies, the benefits of new services, and how to participate in new services is a crucial barrier to the adoption of new services [1,26]. Thus, the promotion of RDT among FPs as well as the public should be improved. On the one hand, as crucial participants in China’s healthcare system, the public’s attitudes, concerns, and emotional support are key factors in determining the future development of RDT services provided by FPs. The results of our research indicate that patients do not understand or trust the RDT services provided by FPs. Therefore, the government, primary medical institutions, the healthcare industry, and FPs should create an atmosphere of public opinion that supports RDT and explains what FP-provided RDT services are, the benefits of such services, and detailed steps for how to use them. On the other hand, as the providers of RDT services, FPs’ knowledge of RDT is also important. A total of 63.7% of the FPs reported that the promotion of RDT was insufficient. Thus, related policies should be introduced to FPs because service users who are more knowledgeable can attract new users [6].

(2) Optimizing relevant laws and policies

In line with most previous studies, our findings suggest that the lack of policy support was one of the greatest barriers to FPs’ use of RDT [12,25,26]. A total of 53.8% of the FPs reported that insufficient and unstandardized laws and regulations, a lack of policy support (58.5%), and a lack of norms (46.9%) were obstacles preventing them from providing RDT services. We propose the following measures to solve this problem. First, the government should actively introduce relevant policies to encourage and guide medical institutions to increase investments in information technology construction and provide policy and financial support. Second, the rights and responsibilities of FPs regarding the provision of RDT services should be clarified. Finally, an incentive mechanism should be introduced to encourage FPs to use RDT. In summary, as the leading force in establishing and operating contract services, the government has an obligation to ensure the smooth progress of RDT services, guarantee their fairness and accessibility, and implement policies regarding RDT [26].

(3) Providing technical support for FPs to use RDT

As shown in our research, insufficient facilities in medical institutions (61.7%) constitute one of the greatest problems for FPs regarding China’s RDT service system. Factors such as the workflow of RDT are complicated, and the lack of technical support is a major barrier to FPs’ use of RDT. Therefore, actionable measures to provide technical support for FPs should be proposed. First, the study revealed that for FPs, especially nonusers, a lack of equipment is the greatest obstacle to their use of RDT. Therefore, the government should improve the informatization level of medical equipment in grassroots hospitals, especially community hospitals and rural hospitals, by updating and upgrading hospital hardware facilities, including improving the internet access speed and the construction of a unified information platform for medical data storage, transmission, and sharing [12,26]. Second, the design and use of medical information systems should be optimized. Attention should be given to the user experience, and the system interface should be concise, clear, and convenient to operate to improve the efficiency and satisfaction of medical staff when using the system. Finally, to provide FPs with relevant courses and training, the information literacy and skills of doctors should be improved through training and academic exchanges to enable them to master the application of information technology methods and skills.

### 4.2. Limitations and Future Developments

There are several limitations in this study, which may inspire future studies. First, the sample in this study included provincial administrative regions across China, but not all provinces were covered; meanwhile, the distribution of samples among provinces was uneven. In the future, research including more FPSs and covering all provinces of the country could be carried out to make the research more nationally representative. Second, the relevance of this research is limited to the RDT behaviors of a specific population, namely, FPs. Thus, the results, findings, and conclusions of this research are not generalizable to other groups, such as patients. As important participants in RDT services, patients may have different aims and needs. As a result, studies adopting the patient perspective could lead to different conclusions. Thus, future studies could focus on patients to compensate for the limitations of the current research. Third, this study discusses the differences in FPs’ use of RDT only from the perspective of their demographic characteristics. In future studies, more factors can be included to explore the factors that affect FPs’ use of RDT. Moreover, some important barriers that may hinder FPs from providing RDT services, such as risk barriers (including privacy and medical risks), tradition barriers, and policy and regulation barriers, were not discussed in this study. In the future, on the one hand, innovation resistance theory (IRT) could be employed to systematically identify barriers that may hinder FPs from providing RDT services; on the other hand, interviews and open-ended questions could be employed to obtain more detailed and context-rich information on barriers that may hinder FPs from providing RDT services. Our sampling method was based on WeChat. Although WeChat has almost 1.38 billion monthly active users [36], there are some people who rarely use WeChat or surf the internet. The responses of web-based surveys may be more inclined to be from those already using internet-based health tools; therefore, certain selection biases were inevitable. In the future, a more balanced sample including those who are less engaged with digital tools should be covered to make the research more representative. Finally, quantitative research has certain limitations: questionnaire surveys are unable to collect descriptive and rich viewpoints, and it is difficult to capture respondents’ personalized experiences and nuances, which limits the depth of the research. Thus, more comprehensive research could be conducted in the future by combining questionnaire investigations, interviews, and open-ended questions to obtain a deeper understanding of FPs’ attitudes towards, challenges in, and motivations regarding the use of digital tools.

## 5. Conclusions

In summary, a series of policies have been proposed by the Chinese government to encourage FPs to use RDT, which illustrates the importance of this issue. Overall, although the FPs’ usage rate and frequency of use of RDT were low and FPs still faced many obstacles in providing internet-based services for patients, on the whole, FPs had a positive attitude toward RDT. The majority of FPs demonstrated that they could benefit from using RDT, and they expressed a growing interest in the use of RDT. The findings of this research can improve policymakers’ understanding of FPs’ use of, barriers to, and attitudes toward RDT. Our findings also provide suggestions for optimizing policies related to FPs and internet-based services.

## Figures and Tables

**Table 1:** Characteristics of the participants.

Characteristic	Respondents
**Gender, n (%)**	n = 682
Male	293 (43.0%)
Female	389 (57.0%)
**Age, years (median)**	37 (31, 45)
**General practitioner (GP), n (%)**	n = 682
No	281 (41.2%)
Yes	401 (58.8%)
**Educational level, n (%)**	n = 682
Junior college degree or lower	278 (40.8%)
Bachelor’s degree	338 (49.6%)
Master’s degree or higher	66 (9.7%)
**Hospital types ^a^, n (%)**	n = 682
Level 3 hospitals Level 2 hospitals	31 (4.5%) 59 (8.7%)
Community hospitals	266 (39.0%)
Township hospitals	314 (46.0%)
Others	12 (1.8%)
**Practice years as a health practitioner, n (%)**	12 (5, 22)
**Practice years as a family physician, n (%)**	4 (2, 7)
**Employment form, n (%)**	n = 682
Positions with establishment	312 (45.7%)
Others	370 (54.3%)
**City level ^b^, n (%)**	n = 682
First-tier city	221 (32.4%)
Below first-tier city	461 (67.6%)
**Member of a family physician team, n (%)**	n = 682
Yes	624 (91.5%)
No	58 (8.5%)
**Working hours on weekdays, n (%)**	8 (8, 9)
**Working hours related to FP work on weekdays, n (%)**	4 (3, 6)
**After-tax income ^c^ (USD), monthly, n (%)**	n = 682
Below 280.8	87 (12.8%)
280.8–421.1	269 (39.4%)
421.2–842.4	185 (27.1%)
842.5–1123.2	71 (10.4%)
1123.3–1404.0	46 (6.7%)
1404.2 or above	24 (3.5%)
**Income related to FP work monthly ^d^ (USD), monthly, n (%)**	n = 682
Below 140.4	296 (43.4%)
140.4–280.7	197 (28.9%)
280.8–421.1	115 (16.9%)
421.2–701.9	57 (8.4%)
702.1 or above	17 (2.5%)
**Have you ever participated in RDT services? n (%)**	n = 682
Yes	435 (63.8%)
No	247 (36.2%)
**Frequency of participating in RDT, n (%)**	n = 435
High frequency (>7 times per week)	83 (19.1%)
Low frequency (0–7 times per week)	352 (80.9%)

Notes: ^a^: Level 3 hospitals are those that provide medical and health services across regions, provinces, cities, and even nationwide, which is the highest hospital level in China. They are medical prevention technical centers with comprehensive capabilities in medical treatment, teaching, and research; Level 2 hospitals are regional hospitals that offer medical and health services to several communities. They are the technical centers for regional medical prevention; Community hospitals and township hospitals are the primary health care institutions in China that provide basic medical care, preventive healthcare, and health management services. Among them, township hospitals are an essential part of the rural healthcare system, primarily serving rural residents while also covering special groups and key populations. Community hospitals primarily serve urban community residents. ^b^: First-tier cities usually refer to large metropolises that hold significant positions and exert leading and radiating influences on national political, economic, and other social activities. These cities play a leading and influential role in national social activities such as production, services, finance, innovation, and circulation. In addition, below-first-tier cities refer to cities and regions other than first-tier cities. ^c^: The original questionnaire’s after-tax income ranges are as follows: Below 2000 (RMB) 2000–3999 (RMB); 3000–5999 (RMB); 6000–7999 (RMB); 8000–9999 (RMB); 10,000 or above (RMB). We converted the after-tax income using the 2024 average exchange rate of 1 USD = 7.1217 RMB. ^d^: The original questionnaire’s income related to FP work ranges are as follows: Below 1000 (RMB) 1000–1999 (RMB); 2000–2999 (RMB); 3000–4999 (RMB); 5000 or above (RMB). We converted the after-tax income using the 2024 average exchange rate of 1 USD = 7.1217 RMB.

**Table 2:** Factors hindering FPs from participating in RDTS (n = 682).

Items	Respondents (N = 682)
**The current problems of RDT services provided by family doctors. n (%)**	
They have not been promoted enough.	436 (63.7%)
Traditional, established habits regarding medical treatment are difficult to change.	485 (71.1%)
The popularization of RDT services is slow.	419 (61.4%)
The facilities of medical institutions are insufficient.	421 (61.7%)
It is difficult to guarantee the quantity and quality of FP.	343 (50.3%)
The related laws and regulations are insufficient and still not standardized.	367 (53.8%)
**Factors that prevent you from participating in RDTS. n (%)**	
Lack of ability.	325 (47.7%)
Work overload.	314 (46.0%)
Lack of policy support.	399 (58.5%)
The technology for RDT services is not mature.	377 (55.3%)
Lack of norms for RDT services.	320 (46.9%)
The workflow of RDT services is complicated.	289 (42.4%)
RDT is not effective.	270 (39.6%)
The safety of RDT services cannot be guaranteed.	252 (37.0%)
Medical consortium cannot provide timely technical support.	255 (37.4%)
Residents do not understand RDT services.	356 (52.5%)
Residents do not trust RDT services.	327 (47.9%)

## Data Availability

The data can be made available from the corresponding authors upon reasonable request.

## Abbreviations

The following abbreviations are used in this manuscript:

FPSs	Family Physician Services
GPs	General Practitioners
PHC	Primary Healthcare
RDT	Remote Diagnosis and Treatment
NHC	National Health Commission
FPs	Family Physicians
